# Cystatin M/E Variant Causes Autosomal Dominant Keratosis Follicularis Spinulosa Decalvans by Dysregulating Cathepsins L and V

**DOI:** 10.3389/fgene.2021.689940

**Published:** 2021-07-12

**Authors:** Katja M. Eckl, Robert Gruber, Louise Brennan, Andrew Marriott, Roswitha Plank, Verena Moosbrugger-Martinz, Stefan Blunder, Anna Schossig, Janine Altmüller, Holger Thiele, Peter Nürnberg, Johannes Zschocke, Hans Christian Hennies, Matthias Schmuth

**Affiliations:** ^1^Department of Biology, Edge Hill University, Ormskirk, United Kingdom; ^2^Department of Dermatology, Medical University of Innsbruck, Innsbruck, Austria; ^3^Department of Biological and Geographical Sciences, University of Huddersfield, Huddersfield, United Kingdom; ^4^Institute of Human Genetics, Medical University of Innsbruck, Innsbruck, Austria; ^5^Cologne Center for Genomics, Faculty of Medicine and Cologne University Hospital, University of Cologne, Cologne, Germany

**Keywords:** keratosis follicularis spinulosa decalvans, congenital disorder of cornification, cicatricial alopecia, cystatin, transglutaminase, epidermal differentiation, cathepsin

## Abstract

Keratosis follicularis spinulosa decalvans (KFSD) is a rare cornification disorder with an X-linked recessive inheritance in most cases. Pathogenic variants causing X-linked KFSD have been described in *MBTPS2*, the gene for a membrane-bound zinc metalloprotease that is involved in the cleavage of sterol regulatory element binding proteins important for the control of transcription. Few families have been identified with an autosomal dominant inheritance of KFSD. We present two members of an Austrian family with a phenotype of KFSD, a mother and her son. The disease was not observed in her parents, pointing to a dominant inheritance with a *de novo* mutation in the index patient. Using whole-exome sequencing, we identified a heterozygous missense variant in *CST6* in DNA samples from the index patient and her affected son. In line with family history, the variant was not present in samples from her parents. *CST6* codes for cystatin M/E, a cysteine protease inhibitor. Patient keratinocytes showed increased expression of cathepsin genes *CTSL* and *CTSV* and reduced expression of transglutaminase genes *TGM1* and *TGM3.* A relative gain of active, cleaved transglutaminases was found in patient keratinocytes compared to control cells. The variant found in *CST6* is expected to affect protein targeting and results in marked disruption of the balance between cystatin M/E activity and its target proteases and eventually transglutaminases 1 and 3. This disturbance leads to an impairment of terminal epidermal differentiation and proper hair shaft formation seen in KFSD.

## Introduction

Keratosis follicularis spinulosa decalvans (KFSD) is a rare congenital cornification disorder, characterised by generalised follicular hyperkeratosis, dry skin, progressive cicatricial alopecia mainly on the scalp, facial erythema, folliculitis, and eye symptoms (Bellet et al., 2008; Aten et al., 2010). The majority of KFSD cases follows an X-linked inheritance pattern (KFSDX, MIM 308800), however, genetic heterogeneity of KFSD has been described. Male-to-male transition and a number of pedigrees with supposed autosomal dominant inheritance have been identified (MIM 612843) (Oosterwijk et al., 1997; Bellet et al., 2008; Castori et al., 2009).

Cases of KFSDX are associated with pathogenic variants in MBTPS2 located on Xp22.12 (Aten et al., 2010). MBTPS2 encodes a membrane bound transcription factor protease, also known as site-2 protease (S2P). This zinc metalloprotease cleaves sterol regulatory element-binding proteins (SREBPs), which act as transcription factors regulating several genes involved in the cholesterol metabolism. Interestingly, pathogenic variants in MBTPS2 were also identified in patients with IFAP syndrome (MIM 308205), a rare genodermatosis characterised by ichthyosis follicularis, atrichia, and photophobia (Oeffner et al., 2009; Mégarbané and Mégarbané, 2011).

In a female patient with KFSD and her affected son, we have now identified a pathogenic variant in *CST6* on chromosome 11q13, the gene encoding cystatin M/E. Cystatins act as cystein protease inhibitors, especially as inhibitors of cathepsins. They consist of three families and cystatin M/E is a type 2 cystatin that is involved in the regulation of epidermal barrier function and differentiation of epidermal keratinocytes (Zeeuwen et al., 2001). Activities of cystatin M/E are mediated by cathepsins D, L, and V and legumain, which are then involved in the regulation of activities of transglutaminases 1 and 3. Transglutaminases can cross-link proteins and are crucially involved in the formation of cornified cell envelopes in the epidermal stratum corneum (Zeeuwen et al., 2004, 2010; Brocklehurst and Philpott, 2013). None of the parents of the affected mother showed any signs of KFSD and the variant in *CST6* was not found in their samples, indicating autosomal dominant inheritance with a causative *de novo* variant in the index patient.

## Patients and Methods

### Probands

Permission for the project was received from the Ethics Committees of the Medical University of Innsbruck, Austria (UN4501), the University Research Ethics Sub-Committee at Edge Hill University (URESC16-KE1), and the School Research Ethics and Integrity Committee at the University of Huddersfield (SAS-SREIC 4.1.19-13). Samples were collected after written informed consent had been granted. The adult female index patient (IK-II/1) provided EDTA blood, scalp hair with roots and a skin punch biopsy. Both parents (IK-I/1 and IK-I/2) of patient IK-II/1 and her sons (IK-III/1 and IK-III/2) provided EDTA blood.

### DNA Extraction and Sequencing

DNA was extracted from blood samples with standard methods. For exome sequencing, 3 μg of DNA was fragmented using sonication technology (Diagenode, Seraing, Belgium). The fragments were end-repaired and adaptor-ligated. After size selection, the library was subjected to enrichment using the Nimblegen SeqCap EZ Human Exome Library v2.0 (Roche, Madison, WI, United States) enrichment kit and sequenced using the Illumina HiSeq 2500 instrument (Illumina, San Diego, CA, United States). Data analysis of filter-passed reads was done with BWA-short in combination with GATK and SAMTOOLS as implemented in Varbank (Cologne Center for Genomics). Scripts were applied for filtering against dbSNP, the 1000 Genomes Project and an in-house database of exome variants. Search for genotypes incompatible with Mendelian inheritance was done using DeNovoGear (Ramu et al., 2013). Further criteria for variant selection were coverage of more than six reads, minimal quality score of 10, minor allele frequency <1%. For Sanger sequencing, exons were PCR amplified and products directly sequenced using the BigDye Terminator v.1.1 Cycle Sequencing Kit (Applied Biosystems, Foster City, CA, United States) on a 3730 DNA Analyzer (Applied Biosystems).

### Isolation of Primary Skin Cells

Keratinocytes and fibroblasts were isolated from the index patient’s skin punch biopsy sample or plastic surgery surplus skin samples as described previously (Eckl et al., 2011). Keratinocytes were cultured in medium KCM (Leigh and Watt, 1994) on a 3T3 feeder cell layer; fibroblasts were grown in DMEM containing 10% FCS and supplemented with 2 mM glutamine, 100 IU/ml penicillin, and 100 μg/ml streptomycin (all Gibco, Thermo Fisher Scientific, Vienna, Austria). Gene expression studies, ICC and Western blotting were obtained from freezing feeder-based normal human epidermal keratinocytes (NHEK) and re-plating cells in a defined serum-free medium (KGM, Lonza, Basel, Switzerland) in the absence of feeder cells 24 to 48 h later. Additional skin samples from the patient or her affected son for further experiments were not available.

### *In vitro* Differentiation of Keratinocytes

Patient and control keratinocytes were plated at 4,000 cells per cm^2^ in KGM (Lonza, Basel, Switzerland) in the absence of feeder cells and incubated at 37°C, 95% humidity, 5% CO_2_ until 90% confluent (5 days). To induce terminal epidermal differentiation, cells were grown with KGM supplemented with 1.15 μl/ml of a 1M CaCl_2_ solution. Cells were harvested and pelleted at days 3, 6, and 9. All experiments were conducted in biological and technical triplicates.

### Quantitative PCR

RNA was extracted from feeder-free keratinocyte cultures (RNeasy Midi Kit, Qiagen, Hilden, Germany) including DNase digestion as described by the manufacturer. After normalisation to 1,000 ng/μl, cDNA was synthesised using the High-Capacity RNA-to-cDNA^TM^ Kit (Thermo Fisher Scientific, Vienna, Austria) following the manufacturer’s instructions with 1 μg RNA per reaction. Produced cDNA was used in the following qPCR reactions using pre-designed Taqman probes (Supplementary Table 1) and the Fast Universal PCR Master Mix (Thermo Fisher Scientific, Vienna, Austria) on a QuantStudio 5 Real-Time PCR system (Thermo Fisher Scientific, Vienna, Austria) using 1 μl of a 1:20 dilution of the cDNA for each reaction in a total volume of 10 μl. Data were analysed using the ΔΔ*C*_t_ method (Schmittgen and Livak, 2008) with *GAPDH*, 18S RNA and *HPRT1* as housekeeping genes for normalisation.

### Western Blotting

Cell pellets were resuspended in RIPA buffer (Sigma Aldrich, Munich, German) supplemented with proteinase inhibitor cocktail (final concentration 1:100, Sigma Aldrich, Munich, Germany). Cells were thoroughly vortexed at high speed and subjected to several freeze-thaw cycles at −20°C before samples were centrifuged at 4°C for 10 min at 8,000×*g*. The supernatant containing the protein fraction was transferred to fresh tubes, aliquotted and stored at −80°C for later use for standard protocols for SDS page Western blotting with antibodies listed in Supplementary Table 2.

### Immunocytochemistry

Keratinocytes from patient IK-II/1 and a matched control donor were cultured on poly-L-lysine coated glass coverslips, fixed with ice-cold methanol and stored at −20°C until further use. Cells were washed once with DPBS, permeabilised with PBS containing 1% Triton X-100 in 1% BSA for 10 min at RT followed by 2 h blocking with 10% FCS/3% BSA/DPBS at RT (FCS, DPBS w/o Mg^2+^, Ca^2+^ from Gibco, Thermo Fisher Scientific; BSA from Carl Roth, Karlsruhe, Germany; Triton X-100 from Sigma Aldrich). Primary antibodies were applied over night at 4°C followed by washing steps with DBPS containing 1% BSA. After incubation with fluorescence-labelled secondary antibodies in the dark for 2 h at RT, cells were washed once, nuclear counterstain (DAPI, Sigma Aldrich) was incubated for 5 min, followed by washing steps with DPBS/BSA, DPBS, and distilled water. Cells on coverslips were mounted on slides using a water-soluble mounting medium (Fluoromount Mounting Medium, Sigma Aldrich). Results were visualised on a DMi8 fluorescence microscope (Leica, Wetzlar, Germany). Antibodies and dilutions are listed in Supplementary Table 2.

### Immunohistochemistry

A quarter of the index patient’s skin biopsy was paraffin embedded and used for standard IHC/P analysis with primary and secondary antibodies listed in Supplementary Table 2. A second quarter of the skin biopsy was snap frozen and used for IHC/F, antibodies listed in Supplementary Table 2.

### Statistical Analysis

One-way analysis of variance (ANOVA) with Tukey’s correction for multiple comparisons, or Dunnet’s correction for multiple comparisons against a single control, were used to assess statistically significant differences within data sets (GraphPad Prism v8, GraphPad Software Inc., La Jolla, CA, United States), and *p* values were determined using Student’s *t*-test.

## Results

### Patients With Keratosis Follicularis Spinulosa Decalvans

**FIGURE 1:**
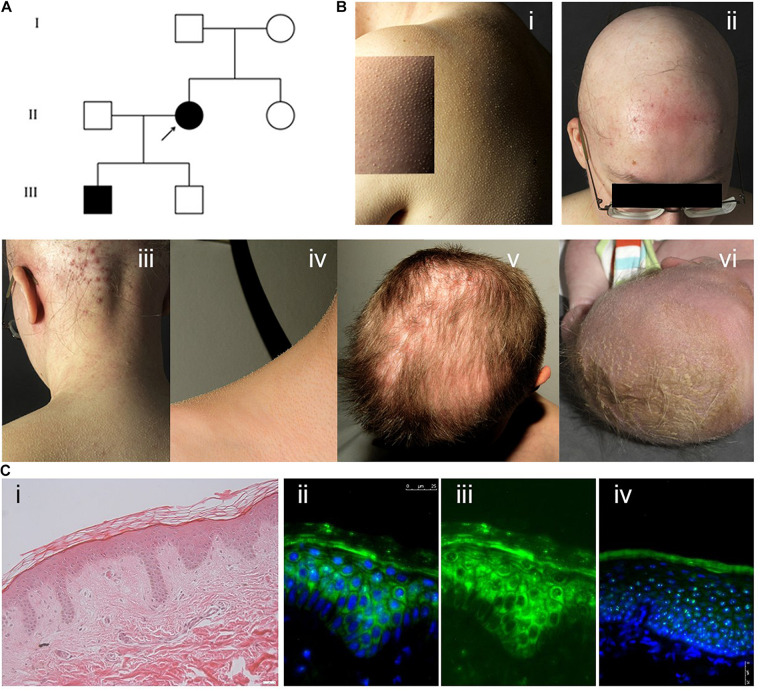
**(A)** Pedigree showing the index patient II/2 and her affected son III/1. The parents of the index patient and her second son were skin-healthy. **(B) (i)** Generalised follicular hyperkeratosis and xerosis in the index patient. **(ii)** Scarring alopecia and folliculitis on the scalp, sparse eyebrows and eyelashes in the index patient. **(iii)** Marked occipital folliculitis in the index patient. **(iv)** Generalised follicular hyperkeratosis in the index patient’s first son (IK-III/1). **(v)** Cicatricial alopecia on the parietal scalp in patient IK-III/1. **(vi)** Severe cradle cap and sparse eyebrows in patient IK-III/1 at the age of 2 weeks. **(C) (i)** H&E staining of paraffin sections of the index patient skin sample showing mild hyperkeratosis, acanthosis with elongated rete ridges and perifollicular fibrosis. **(ii–iv)** Cystatin M/E expression in paraffin-embedded skin samples from patient IK-II/1 **(ii,iii)** and healthy control person **(iv)**. Cystatin M/E expression is confined to the upper layers of the viable epidermis in control samples but distributed over all suprabasal layers in the patient sample. **(ii,iv)** Merged images with DAPI staining, **(iii)** cystatin M/E staining only. Magnification bar represents 25 μm.

The index patient (IK-II/2) of an Austrian family with KFSD (Figure 1A), a 31-year-old woman, presented with generalised follicular hyperkeratosis, dry skin, scarring alopecia on the scalp, sparse eyebrows and eyelashes and occipital folliculitis (Figure 1B). In addition, she showed hypohidrosis and myopia with astigmatism. Her 4-year-old son (IK-III/1) exhibited severe cradle cap postnatally and a bacterial conjunctivitis, but later he developed a skin phenotype like his mother, with sparse hair and lack of eyelashes. His cicatricial alopecia was limited to the parietal scalp (Figures 1B/v,vi). Mother and son reported pruritus in particular in areas of cicatricial alopecia. The other family members did not exhibit any cutaneous symptoms. A medical check-up of both affected individuals did not reveal signs of physical or intellectual disabilities or an involvement of other organs. H&E staining of the paraffin-embedded skin sample of the index patient showed acanthosis with elongated rete ridges and perifollicular fibrosis (Figure 1C/i).

### Mutation Analysis

After variants in *MBTPS2* had been ruled out as a cause for the disease by direct sequencing, DNA samples from the index patient and her parents were further analysed by whole-exome sequencing. The sequencing achieved a mean coverage of 92×, 103×, and 122×, respectively, with 10× coverage of at least 97% of the targets. Taking co-segregation into consideration, missense variants in three genes were identified (*CST6*, *EMSY*, and *PHB*). Focusing on prominent gene expression in keratinocytes and hair follicles (Fagerberg et al., 2014; Uhlén et al., 2015), only one variant was identified, c.65T > C in *CST6* (NM_001323.4; Genbank gene ID 1474). The variant is predicted to lead to the replacement of leucine by proline at residue 22 of cystatin M/E [p.(Leu22Pro)]. The affected residue Leu22 is highly conserved, showing 70% identity in an alignment of cystatin M/E orthologs with HMMER 3 in 150 species and 86% in an iterated psi-BLAST alignment (111 species). The physicochemical difference is moderate between Leu and Pro (Grantham score 98), however, SIFT and PolyPhen predicted the variant as deleterious (SIFT score 0) and probably damaging (PolyPhen-2 score 0.927), respectively. The variant has not been identified in sequencing projects (gnomAD, 1000 genomes). Sanger sequencing in samples from the index patient, her parents (IK-I/1, 2) and sons (IK-III/1, 2) showed absence of this variant in the parents, confirming *de novo* occurrence, and co-segregation with the phenotype in the sons (Supplementary Figure 1).

### Gene Expression Analysis

**FIGURE 2:**
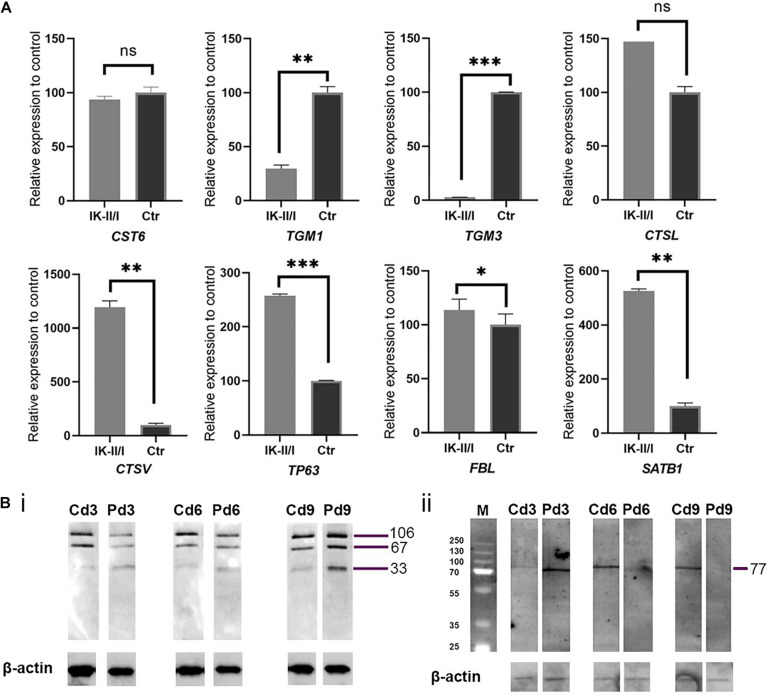
**(A)** Analysis of relative expression for genes *CST6*, *TGM1*, *TGM3*, *CTSL*, *CTSV*, *TP63*, *FBL*, and *SATB1* comparing RNA from patient and control keratinocytes grown feeder-free under high Ca^2+^ conditions and normalised to 18S RNA and housekeeping genes *GAPDH* and *HPRT1*. All gene expression studies were conducted in biological triplicates. Expression of *TGM1* and *TGM3* was significantly reduced in patient keratinocytes. Expression of cathepsin genes was strongly upregulated. ns, not significant; ^∗^*p* ≤ 0.05; ^∗∗^*p* ≤ 0.01; ^∗∗∗^*p* ≤ 0.001. **(B)** Western blotting analysis of (i) Tgase-1 and (ii) Tgase-3 in patient (P) and matched control (C) keratinocytes. Keratinocytes were cultured for 3, 6, and 9 days under high Ca^2+^ conditions. β-actin was used as a loading control. (i) Tgase-1 was only weakly expressed in day 3 and day 6 patient cells but stronger after 9 days, with a prominent 33 kDa band indicating an increased fractionising of Tgase-1 in patient cells. (ii) Full-length Tgase-3 was not detectable in day 6 or day 9 patient samples, suggesting either a lack of Tgase-3 or a near-to-complete fractionising of the 77 kDa zymogen to its active 33/47 kDa fragments, which were not detectable with this analysis.

*CST6* encodes cystatin M/E, which antagonises lysosomal cysteine proteases. IHC/P analysis of patient skin samples showed a broadened expression of cystatin M/E (Figures 1C/ii–iv). Quantitative analysis of *CST6* transcripts in patient keratinocytes cultured under feeder-free, high Ca^2+^ (1.15 mM) conditions showed a slight reduction as compared to control samples (Figure 2). We investigated the expression of cathepsins directly regulated by cystatin M/E in terminally differentiating keratinocytes. We found a significant increase in expression of *CTSL* and *CTSV* in patient cells as compared to matched controls (Figure 2A); in contrast, there was no significant change in *LGMN* expression (not shown). We did not detect any differences in the distribution of transglutaminase 1 (Tgase-1) in paraffin-embedded skin sections from the patient specimen (Supplementary Figure 2); however, *TGM1* expression was strongly reduced in patient cells compared to healthy controls (Figure 2A). Importantly, *TGM3* expression was also markedly reduced in patient keratinocytes (Figure 2A).

**FIGURE 3:**
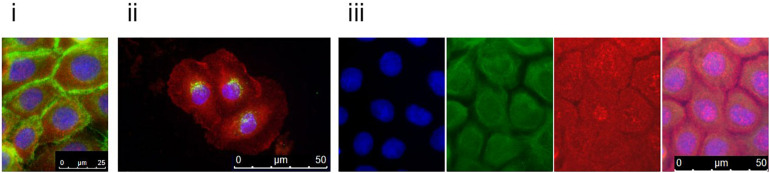
Patient keratinocytes were grown on coverslips and double-stained with antibodies against cystatin M/E (red) and **(i)** E-cadherin, **(ii)** GM130, or **(iii)** keratin 14 (green). Counterstaining was done with DAPI. Patient keratinocytes clearly expressed cystatin M/E abundantly in the cytosol. For the keratin 14 double-staining, DAPI (blue), keratin 14 (green), and cystatin M/E (red) are shown separately and merged. Magnification bars represent 25 μm **(i)** or 50 μm **(ii,iii)**.

To follow up on these findings, we investigated Tgase-1 and Tgase-3 by Western blot analysis. Tgase-1 was weak in patient samples after 3 and 6 days of differentiation but strong on day 9 with a prominent 33 kDa band, indicating the smaller cytosolic fragment and showing an increased fractionising and activation of Tgase-1 in patient cells (Figure 2B). Full-length Tgase-3 was present at day 3 of differentiation but not detectable in day 6 or day 9 patient samples, suggesting either a lack of Tgase-3 at days 6 and 9 or, more probably, a nearly complete digestion of the 77 kDa zymogen to its active 33/47 kDa fragments, which were not detectable with this analysis. Variants in other protease inhibitors have been described to cause cell-cell adhesion deficits (Blaydon et al., 2011). Therefore, we assessed the distribution of E-cadherin in patient cells (Figure 3) and investigated co-localisations of cystatin M/E with GM130 and keratin 14, respectively (Figures 3/ii,iii). These experiments showed a strong presence of cystatin M/E in the cytoplasm but no signs of disturbed keratinocyte adhesion or cytoskeleton. Additional qPCR analysis for *TP63* and *SATB1*, which are involved in tissue-specific chromatin remodelling and the control of epidermal differentiation, showed an upregulation in patient cells (Figure 2A).

## Discussion

Cystatin M/E is a type II cystatin that is both glycosylated and phosphorylated and secreted into the extracellular space. It is synthesised as a pre-protein with an N-terminal signal sequence. While the extent of the signal peptide is not completely clear (Ni et al., 1997; Sotiropoulou et al., 1997), hydrophobicity analysis pointed to a 22-residue signal peptide. This is underlined by the fact that cystatin M/E has a proline residue at position 23 and several arginine as well as other charged residues immediately thereafter. This means that alterations at Leu22, the residue affected here, would most probably affect the processing of the pre-protein and thus the targeting of active cystatin M/E. Preliminary experiments indicated a marked location of mutated cystatin M/E in the cytosol. Similarly, a substitution of alanine by threonine at position 25 in cystatin C, the last of the N-terminal hydrophobic residues, was associated with an increased susceptibility to age-related macular degeneration (Zurdel et al., 2002). Recombinant altered cystatin C was erroneously targeted to mitochondria and also found throughout the cytoplasm and nucleus of retinal cells (Paraoan et al., 2004); secretion of a threonine variant fusion protein was reduced by approximately 50% compared with wild-type protein.

Cystatin M/E inhibits legumain and cathepsins L and V (Zeeuwen et al., 2007) and acts as a tumour suppressor (Briggs et al., 2010). Cathepsins are cysteine proteases essential for proper cornification and hair shaft development and required for the activation and processing of a variety of epidermal proteins, amongst them Tgase-1 and Tgase-3 (Cheng et al., 2009; Zeeuwen et al., 2010). Increased expression of *CTSV* and *CTSL* might indicate a feedback regulation following the cytosolic activity of mutant inhibitor cystatin M/E. Dysregulation of Tgase 1 and Tgase 3 can account for disturbed terminal differentiation of keratinocytes, hyperkeratosis and hair shaft anomalies (Candi et al., 2005; Eckhart et al., 2013). SATB1 and p63, transcription and remodelling factors encoded by *SATB1* and *TP63*, may contribute to the phenotype of hyperplasia and hyperkeratosis.

Recently, Van den Bogaard and colleagues described a family with autosomal recessive hypotrichosis and dry skin caused by homozygous nonsense variant c.361C > T in *CST6*, resulting in the expression of truncated and only partly functional cystatin M/E that lacked the inhibitory function for cathepsins V and L and legumain (van den Bogaard et al., 2019). Heterozygous family members did not show any related symptoms. In contrast, the heterozygous variant described here is expected to result in a gain of function. Whereas patients in the above family presented with hypotrichosis of scalp and body hair, the patients presented here with autosomal dominant KFSD due to a *CST6* variant display follicular hyperkeratosis and scarring. Individuals from both families showed sparse eyelashes and hypohidrosis. These phenotypic differences indicate alternative pathomechanisms. Whereas *TGM1* expression was increased in samples from patients with homozygous loss-of-function variants in *CST6*, we observed a decline of *TGM1* expression in samples with the dominant *CST6* variant. Further studies will be needed to determine the outcome of the altered *CST6* mutant in more details and to substantiate its effects on cathepsin expression and activity. A significantly altered activation pattern of transglutaminases in keratinocytes from our patients indicates disturbance of cornification associated with substantially increased cathepsin levels.

## Data Availability Statement

The datasets presented in this article are not readily available because for legal reasons, individual genomic DNA data cannot be made available.

## Ethics Statement

The studies involving human participants were reviewed and approved by the Ethics Committees of the Medical University of Innsbruck, Austria (UN4501), the University Research Ethics Sub-Committee at Edge Hill University (URESC16-KE1), the School Research Ethics and Integrity Committee at the University of Huddersfield (SAS-SREIC 4.1.19-13). Written informed consent to participate in this study was provided by the participants or the participants’ legal guardian/next of kin. Written informed consent was obtained from the individuals and minors’ legal guardian/next of kin, for the publication of any potentially identifiable images or data included in this article.

## Author Contributions

KE, RG, HH, and MS: conceptualization and methodology. HT: software. JA: validation and formal analysis. KE, RG, LB, AM, RP, VM-M, SB, and AS: investigation. PN, JZ, HH, and MS: resources. JA and HT: data curation. KE and RG: writing – original draft preparation. KE, RG, and HH: visualization. HH and MS: supervision. KE, HH, and MS: funding acquisition. All authors writing, review, and editing.

## Conflict of Interest

The authors declare that the research was conducted in the absence of any commercial or financial relationships that could be construed as a potential conflict of interest.
