# Supplementary material for: Cystatin M/E Variant Causes Autosomal Dominant Keratosis Follicularis Spinulosa Decalvans by Dysregulating Cathepsins L and V
Source: Front Genet. 2021 Jul 12;12:689940. doi: 10.3389/fgene.2021.689940 (PMC8312243; doi:10.3389/fgene.2021.689940)
Supplement: Supplementary file 1 [file Data_Sheet_1.PDF]

Cystatin M/E gain-of-function variant causes autosomal dominant keratosis follicularis spinulosa decalvans by dysregulating cathepsins L and V

Katja M. Eckl[1]†, Robert Gruber[2]†, Louise Brennan[1], Andrew Marriott[1], Roswitha Plank[3,4], Verena Moosbrugger-Martinz[2], Stefan Blunder[2], Anna Schossig[4], Janine Altmüller[5], Holger Thiele[5], Peter Nürnberg[5], Johannes Zschocke[4], Hans Christian Hennies[3,5]*, Matthias Schmuth[2]*

[1]Department of Biology, Edge Hill University, Ormskirk, UK

[2]Department of Dermatology, Medical University of Innsbruck, Innsbruck, Austria

[3]Department of Biological and Geographical Sciences, University of Huddersfield, Huddersfield, UK

[4]Institute of Human Genetics, Medical University of Innsbruck, Innsbruck, Austria

[5]Cologne Center for Genomics, Faculty of Medicine and Cologne University Hospital, University of Cologne, Köln, Germany

† Equal contribution

* Correspondence:

Hans Christian Hennies
h.c.hennies@hud.ac.uk

Matthias Schmuth
matthias.schmuth@i-med.ac.at

Supplementary Material

SUPPLEMENTARY FIGURES AND TABLES

Supplementary Figures

FIGURE S1

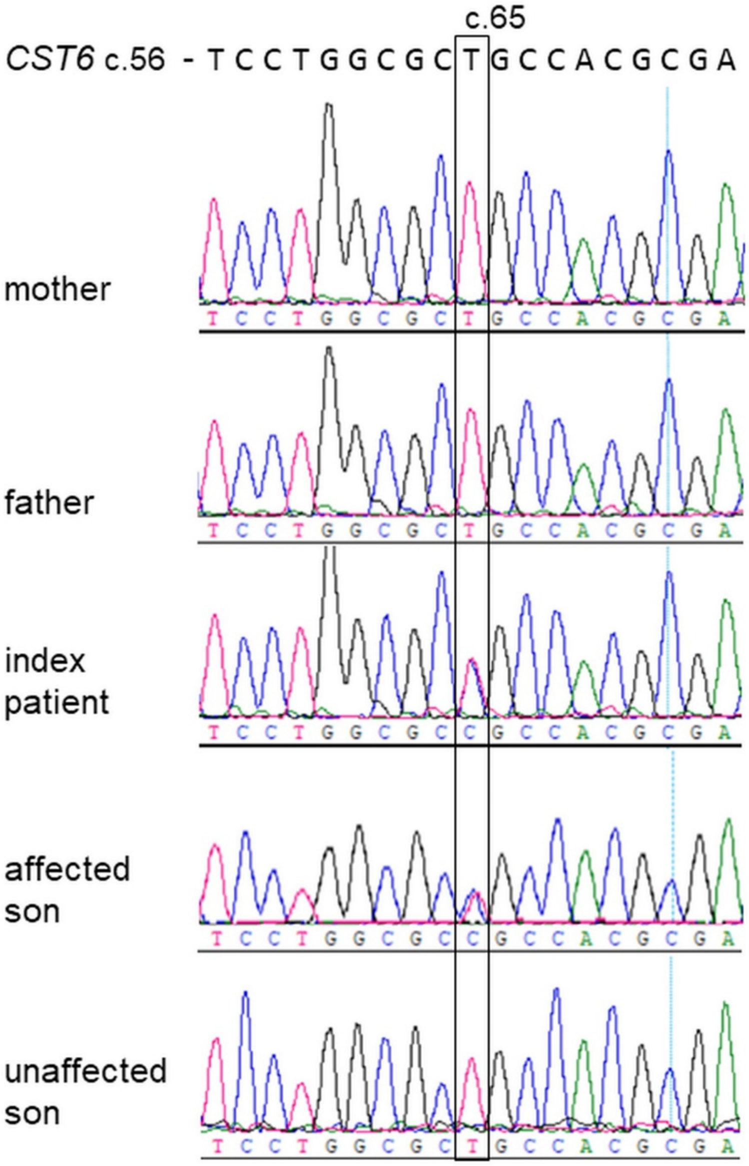

Supplementary Figure S1. Sanger sequencing in DNA samples from the family confirmed the heterozygous variant c.65T>C in *CST6* in the patient. The variant was not seen in the parents of the patient, showing a *de novo* variant. Co-segregation was found and the variant was also present in the patient’s affected son but not in her unaffected son.

FIGURE S2

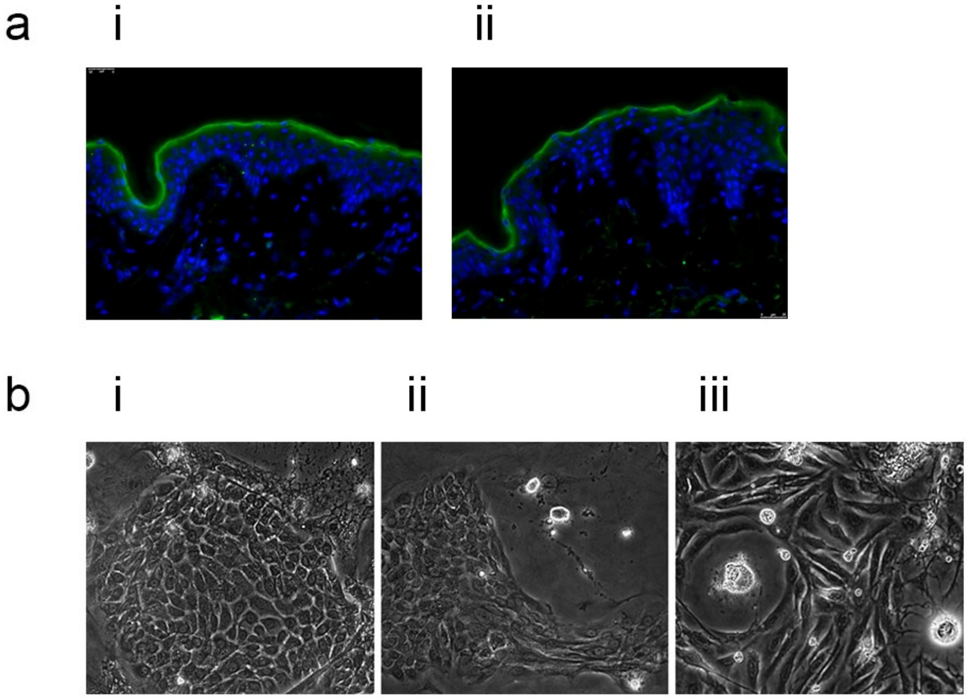

Supplementary Figure S2. (a) Detection of Tgase-1 expression in paraffin-embedded skin samples from (i) patient IK-II/1 and (ii) a healthy control person. Tgase-1 expression was detected in the stratum granulosum and stratum corneum in both samples. Counter stain was performed with DAPI (nuclear stain). Magnification bar represents 25 µm.

(b) Keratinocytes from index patient IK-II/1 exhibited an unusual growth and morphological phenotype in as early as passage 4. Keratinocytes showed a typical colony forming appearance in early passages (i), but changed morphology and gene expression spontaneously both in feeder-based and feeder-free conditions from passage 4 (ii, iii). Cells remained proliferative but grew very slowly.

Supplementary Tables

Table S1. Antibodies for Western blotting, immunocytochemistry and immunohistochemistry

Antibodies	Company	Order ID	Host	Dilution	Application
Anti-Tgase-1	Santa Cruz	sc-166467	Mouse (MC)	1:100	WB, IHC/P
Anti-Tgase-3	R&D	AF4604	Sheep (PC)	1:400	WB
Anti-cystatin M/E	Sigma Aldrich	HPA044963	Rabbit (PC)	1:500	IHC/P
Anti-cystatin M/E	Novus Biologicals	28730002	Rabbit (PC)	1:200	IHC/F<br>IHC/P
Anti-GM130	R&D	610822	Mouse (MC)	1:50	ICC
Anti-β-actin	Abcam	ab6276	Mouse (MC)	1:5000	WB
Anti-E-cadherin	R&D	562869	Mouse (MC)	1:50	ICC
Secondary antibodies	Thermo Fisher Scientific	Alexa-488 or TxR labelled	Depending on primary antibody	1:400	ICC, IHC/P
		HRP-coupled		1:5000	WB

Table S2: TaqMan assays for qPCR (Thermo Fisher Scientific)

**Gene**	**Assay ID**	**Target / Housekeeping**
FLB	Hs01070449_m1	Target
CST6	Hs01012810_g1	Target
CSTB	Hs00947433_m1	Target
CSTV	Hs00952036_m1	Target
TGM3	Hs00162752_m1	Target
TGM1	Hs01070310_m1	Target
CSTL	Hs00964650_m1	Target
TP63	Hs00978339_m1	Target
18S RNA	Hs99999901_s1	Housekeeping
GAPDH	Hs02758991_g1	Housekeeping
HPRT1	Hs99999909_m1	Housekeeping
